# Comparison of En Masse Retraction and Two-Step Retraction Mechanics in Class I Bimaxillary Protrusion: A Retrospective Comparative Study

**DOI:** 10.7759/cureus.112305

**Published:** 2026-07-08

**Authors:** Bhavik Ramanlal Patel, M. Akhilesh, Kommu Pavan, Chidambram S, Srinu Kashana, Jasuma Rai, Adeel A Bajjad

**Affiliations:** 1 Department of Orthodontics, A. J. Institute of Dental Sciences, Mangalore, IND; 2 Department of Orthodontics, Kalavati Dental and Implant Centre, Hyderabad, IND; 3 Department of Orthodontics, Tirumala Institute of Dental Sciences and Research Center, Nizamabad, IND; 4 Department of Orthodontics, JKKN Dental College and Hospital, Komarapalayam, IND; 5 Department of Dentistry, Global Smiles Dental, Indianapolis, USA; 6 Department of Periodontology, K. M. Shah Dental College and Hospital, Sumandeep Vidyapeeth (Deemed to be University), Vadodara, IND; 7 Department of Orthodontics, Kothiwal Dental College and Research Centre, Moradabad, IND

**Keywords:** anchorage, bimaxillary protrusion, orthodontic, orthodontic space closure, orthodontic tooth movement, tooth extraction

## Abstract

Introduction: Bimaxillary protrusion is commonly treated using extraction-based orthodontic therapy, followed by anterior tooth retraction. En masse retraction and two-step retraction are widely used space-closure mechanics; however, differences in treatment efficiency, anchorage preservation, and arch dimensional changes remain. This study aimed to compare the outcomes of en masse retraction and two-step retraction mechanics in patients with Class I bimaxillary protrusion undergoing extraction-based orthodontic treatment.

Materials and methods: This retrospective comparative study evaluated the completed orthodontic records of 100 patients with Class I bimaxillary protrusion who were treated between 2016 and 2021. The patients were classified into two groups: en masse retraction (n = 50) and two-step retraction (n = 50). Measurements were obtained from the pretreatment and post-treatment study models and treatment records. The variables assessed were maxillary incisor retraction, anchorage loss, treatment duration, retraction rate, extraction space closure efficiency, residual extraction space, and transverse arch dimensional changes. Data were analyzed using an independent samples t-test, a chi-square test, and a Mann-Whitney U test. Statistical significance was set at p < 0.05.

Results: Both treatment modalities achieved comparable maxillary incisor (6.84 ± 1.12 mm vs. 6.52 ± 1.08 mm; p = 0.129). The en masse retraction group demonstrated significantly greater anchorage loss, with a first molar mesial drift of 2.18 ± 0.76 mm compared with 1.43 ± 0.61 mm in the two-step retraction group (p < 0.001). Space closure duration was significantly shorter in the en masse group (8.6 ± 1.8 months) than in the two-step group (11.4 ± 2.3 months) (p < 0.001). Extraction space closure efficiency was higher in the en masse group (96.90 ± 2.80%) than in the two-step group (95.40 ± 3.20%) (p = 0.011). Significant reductions in intercanine, intermolar, and interpremolar widths were observed in the en masse group compared with the two-step group (p < 0.001).

Conclusion: Both retraction mechanics were effective in achieving anterior tooth movement. En masse retraction provided faster space closure and greater treatment efficiency but was associated with increased anchorage loss and greater transverse arch constriction. Two-step retraction demonstrated superior anchorage preservation and transverse dimension stability.

## Introduction

Bimaxillary protrusion is a common dentofacial condition characterized by protrusive maxillary and mandibular incisors, increased lip prominence, and convex facial profile. Orthodontic correction of this malocclusion frequently involves the extraction of the first premolars, followed by the retraction of the anterior dentition to improve facial esthetics, lip competence, and occlusal relationships [1-3]. Among the various space closure strategies employed during extraction-based orthodontic treatment, en masse retraction and two-step retraction are the most commonly used approaches [3].

En masse retraction involves simultaneous retraction of the anterior teeth as a single unit after alignment and leveling, whereas two-step retraction involves initial canine retraction followed by retraction of the incisors [3,4]. Both techniques have distinct biomechanical characteristics that may influence treatment efficiency, anchorage preservation, space closure rate, and overall treatment duration.

Although several studies and systematic reviews have reported that en masse retraction is associated with reduced treatment duration and improved anchorage control compared with two-step retraction, the majority of these investigations have evaluated en masse mechanics in conjunction with temporary anchorage devices (TADs) or mini-implants [3,5-7]. The use of skeletal anchorage substantially minimizes anchorage loss and may influence the overall efficiency of space closure [7]. However, mini-implants are not always feasible in routine clinical practice because of additional costs, patient acceptance, and financial constraints, particularly in resource-limited settings. Consequently, many patients continue to be treated with conventional anchorage systems without skeletal reinforcement. Despite their widespread use, there is a paucity of evidence directly comparing conventional en masse retraction and conventional two-step retraction mechanics.

Therefore, the present study aimed to evaluate and compare the treatment outcomes, anchorage preservation, space closure efficiency, and treatment duration associated with these two conventional retraction approaches in patients with Class I bimaxillary protrusion. The objectives were to evaluate maxillary incisor retraction, anchorage loss, treatment duration, retraction rate, extraction space closure efficiency, residual extraction space, transverse arch dimensional changes, and overall treatment outcomes associated with the two treatment modalities.

## Materials and methods

Study design and setting

This retrospective study was conducted in the Department of Orthodontics, Kothiwal Dental College and Research Center, Moradabad, India, using completed orthodontic treatment records. The study was conducted in November 2021 and involved the evaluation of orthodontic records of patients treated between January 2016 and October 2021. As the present investigation was based exclusively on the review of completed orthodontic records and study models with no direct patient involvement or intervention, the requirement for ethical approval and informed consent was waived by the institutional authorities in accordance with the institutional policy governing retrospective record-based studies. All patient information was anonymized before data collection to ensure confidentiality of the data.

Sample size estimation

The sample size was estimated using G*Power software (version 3.1.9.7; Heinrich Heine University, Düsseldorf, Germany). Based on the findings reported by Felemban et al. [8], who evaluated orthodontic space closure and anchorage outcomes between different retraction mechanics, an effect size of 0.57 was obtained. Considering a confidence level of 95% (α = 0.05), statistical power of 80%, and a two-tailed comparison, the minimum required sample size was calculated to be 45 participants per group. To compensate for incomplete records and potential missing data commonly encountered in retrospective investigations, the sample size was increased to 50 patients per group, resulting in a total study sample of 100 participants.

Study sample

Orthodontic records of patients diagnosed with Class I bimaxillary protrusion who had undergone extraction of all four first premolars, followed by fixed appliance therapy, were screened for eligibility. Patients aged 15-30 years with complete pretreatment and post-treatment records, including study models, cephalometric radiographs, treatment progress records, and treatment completion records, were included in the study. Patients with previous orthodontic treatment, craniofacial anomalies, syndromic conditions, orthognathic surgical treatment, congenitally missing permanent teeth other than extraction premolars, incomplete records, or poor-quality study models were excluded from the analysis. Eligible records were categorized into two groups according to the space-closure mechanics used during treatment. Group I comprised patients treated using en masse retraction mechanics, whereas Group II comprised patients treated using two-step retraction mechanics.

Data collection and measurements

Data were collected from archived patient records and study models. Demographic information, treatment duration, and details regarding orthodontic mechanics were obtained from the patient files. Measurements related to anterior tooth retraction, anchorage loss, extraction space closure, and transverse arch dimensions were obtained from the pretreatment and post-treatment study models.

**Figure 1:**
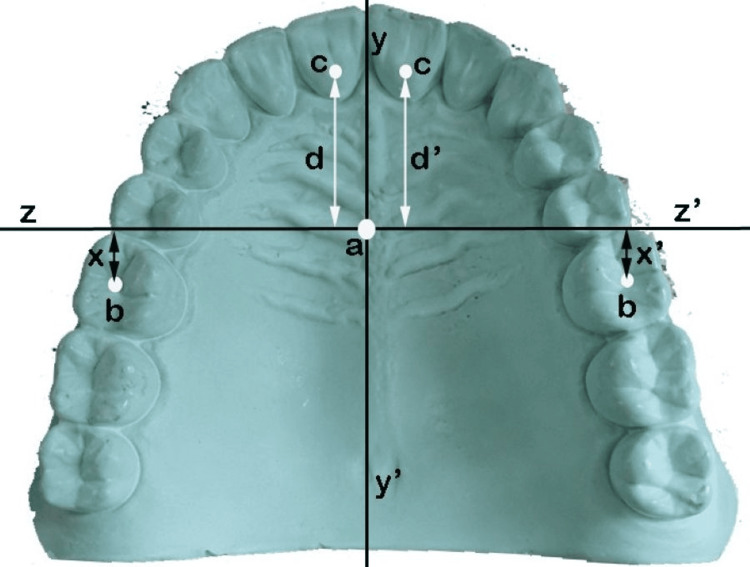
Schematic representation of the reference landmarks and linear measurements used in the study on the study models for assessment of incisor retraction and anchorage loss. a point: a stable point located on the third palatal rugae, b point: a point on the central fossa of the first maxillary molar, C point: a point on the cingulum of the central incisor. The vertical line y–y′ passes through the mid‑palatine raphe. The horizontal line z–z′ passes through the stable point (a). Distances x and x′ represent the perpendicular distances from the central fossa (b) to the horizontal line z–z′ to represent anchorage loss; distances d and d′ represent the perpendicular distances from the cingulum of the central incisor (C) to the horizontal line z–z′ to represent incisor retraction. Original image created by the authors using Canva (Canva Pty Ltd., Sydney, NSW, Australia).

All linear measurements were recorded using a digital Vernier caliper with an accuracy of 0.01 mm. The third palatal rugae region was used as a stable intraoral reference structure to assess maxillary incisor movement and anchorage loss [9]. The amount of maxillary incisor retraction was determined by measuring the distance from the cingulum point (c point) to the horizontal line passing through the third palatal rugae (Z-Z' line). Anchorage loss was assessed by evaluating the mesial movement of the maxillary first molars relative to the reference landmarks (Figure 1).

Treatment duration was calculated from the date of appliance placement to appliance removal, whereas space closure duration was determined from the initiation of retraction mechanics until complete closure of the extraction spaces. The retraction rate was calculated by dividing the amount of tooth movement by the corresponding space closure duration. The residual extraction space was measured at the end of the treatment. Extraction space closure efficiency was calculated using the following formula:

$\text{Extraction space closure efficiency} = \left[ \frac{\text {Available extraction space}-\text{Residual extraction space}}{\text{Available extraction space}} \right]\times 100\%$

**Figure 2:**
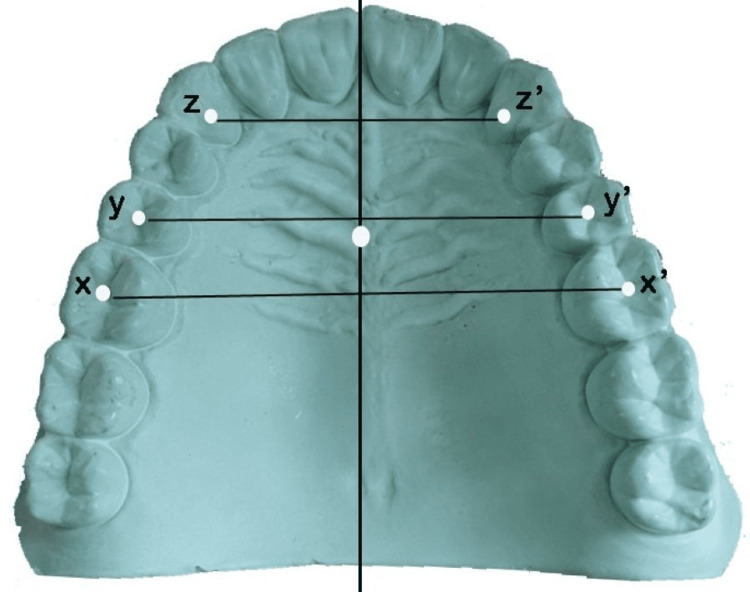
Assessment of transverse arch dimensions using maxillary study models. Intercanine width as assessed from z-z' distance, where z and z' are points on right and left canine fossae, Interpremolar width as assessed from y-y' distance, where y and y' are points on central fossae of right and left second premolars, Intermolar width as assessed from x-x' distance, where x and x' are points on central fossae of right and left first molars. Original image created by the authors using Canva (Canva Pty Ltd., Sydney, NSW, Australia).

Transverse arch dimensions were assessed by measuring intercanine, interpremolar, and intermolar widths before and after treatment (Figure 2).

Calibration and reliability assessment

Prior to commencing the measurements, the principal investigator underwent calibration under the supervision of a senior orthodontist experienced in orthodontic model analysis. To assess measurement reproducibility, 20 randomly selected study models were re-evaluated after a two-week interval without reference to the initial measurements. Intra-examiner reliability was assessed using the intraclass correlation coefficient (ICC) based on a two-way mixed-effects model with absolute agreement. The ICC values ranged from 0.968 to 0.991, indicating excellent reproducibility and consistency of measurements. All reliability coefficients were statistically significant (p < 0.001), confirming minimal measurement error and excellent methodological reliability.

Outcome measures

The primary outcome measures included the amount of maxillary incisor retraction, anchorage loss, and the ratio of incisor retraction to anchorage loss. Secondary outcome measures included treatment duration, space closure duration, retraction rate, overall space closure rate, residual extraction space, extraction space closure efficiency, and changes in transverse arch dimensions, including intercanine, interpremolar, and intermolar widths.

Statistical analysis

All statistical analyses were performed using IBM SPSS Statistics for Windows (version 23.0; IBM Corp., Armonk, New York, USA). Descriptive statistics were expressed as mean ± standard deviation (SD) for continuous variables and frequency with percentages for categorical variables. The normality of the data distribution was assessed using the Shapiro-Wilk test. Intergroup comparisons of normally distributed continuous variables were performed using the independent samples t-test, whereas categorical variables were compared using the chi-square test. A p-value of less than 0.05 was considered statistically significant for all the analyses.

## Results

A total of 100 patients were included in the study, with 50 (50%) patients allocated to the en masse retraction group and 50 (50%) patients allocated to the two-step retraction group. Table 1 presents the demographic and baseline characteristics of the participants. The en masse retraction group comprised 18 (36.0%) men and 32 (64.0%) women, whereas the two-step retraction group included 20 (40.0%) men and 30 (60.0%) women. Class I skeletal patterns were observed in 38 (76.0%) and 36 (72.0 %) patients in the en masse retraction and two-step retraction groups, respectively. No statistically significant differences were observed between the groups in terms of age, sex distribution, skeletal pattern, pretreatment overjet, pretreatment overbite, or total treatment duration (p > 0.05), indicating good baseline comparability.

**Table 1:** Demographic characteristics of the study participants. Data presented as mean ± standard deviation (SD) or n (%), independent samples t-test used for continuous variables, Chi-square test used for categorical variables, p < 0.05 considered statistically significant. SD: standard deviation.

Variable	En masse retraction (n = 50)	Two-step retraction (n = 50)	Test value	p-value
Age (years), Mean ± SD	19.8 ± 3.4	20.2 ± 3.1	t = -0.615	0.528
Sex, Male, n (%)	18 (36.0%)	20 (40.0%)	χ² = 0.170	0.681
Sex, Female, n (%)	32 (64.0%)	30 (60.0%)		
Skeletal pattern, Class I, n (%)	38 (76.0%)	36 (72.0%)	χ² = 0.208	0.643
Skeletal pattern, Mild Class II tendency, n (%)	12 (24.0%)	14 (28.0%)		
Pretreatment overjet (mm), Mean ± SD	5.8 ± 1.2	5.9 ± 1.3	t = -0.400	0.714
Pretreatment overbite (mm), Mean ± SD	2.9 ± 0.8	3.0 ± 0.7	t = -0.665	0.512
Total treatment duration (months), Mean ± SD	28.4 ± 4.6	30.1 ± 5.2	t = -1.731	0.078

Table 2 shows a comparison of anterior retraction and anchorage loss between the two treatment modalities. The amount of maxillary incisor retraction was slightly greater in the en masse retraction group (6.84 ± 1.12 mm) than in the two-step retraction group (6.52 ± 1.08 mm), although the difference was not statistically significant (p = 0.129). However, anchorage loss was significantly greater in the en masse retraction group, with first molar mesial drift measuring 2.18 ± 0.76 mm compared with 1.43 ± 0.61 mm in the two-step retraction group (p < 0.001). Likewise, second premolar mesial drift was significantly higher in the en masse retraction group (1.74 ± 0.65 mm) than in the two-step retraction group (1.12 ± 0.58 mm) (p < 0.001). The retraction-to-anchorage loss ratio was significantly greater in the two-step retraction group (4.56 ± 0.84) than in the en masse retraction group (3.14 ± 0.62) (p < 0.001).

**Table 2:** Comparison of incisor retraction and anchorage loss between the study groups. Data presented as mean ± standard deviation (SD), independent samples t-test used for intergroup comparisons, *p < 0.05 considered statistically significant, SD: standard deviation.

Parameter	En masse retraction (mean ± SD)	Two-step retraction (mean ± SD)	Test value	p-value
Amount of incisor retraction (mm)	6.84 ± 1.12	6.52 ± 1.08	t = 1.454	0.129
Amount of anchorage loss with respect to mesial movement of first molar (mm)	2.18 ± 0.76	1.43 ± 0.61	t = 5.442	<0.001*
Anchorage loss with respect to mesial movement of second premolar (mm)	1.74 ± 0.65	1.12 ± 0.58	t = 5.033	<0.001*
Ratio: incisor retraction/anchorage loss	3.14 ± 0.62	4.56 ± 0.84	t = -9.617	<0.001*

Table 3 summarizes the treatment duration and retraction rate outcomes. The en masse retraction group demonstrated a significantly shorter space closure duration (8.6 ± 1.8 months) than the two-step retraction group (11.4 ± 2.3 months) (p < 0.001). The rate of maxillary incisor retraction was significantly higher in the en masse retraction group (0.82 ± 0.14 mm/month) than that in the two-step retraction group (0.58 ± 0.11 mm/month) (p < 0.001). No significant difference was observed in the leveling and alignment durations between the groups (p = 0.302).

**Table 3:** Comparison of treatment duration and retraction rate between the study groups. Data presented as mean ± standard deviation (SD), independent samples t-test used for intergroup comparisons, *p < 0.05 considered statistically significant, SD: standard deviation.

Parameter	En masse retraction (mean ± SD)	Two-step retraction (mean ± SD)	Test value	p-value
Space closure duration (months)	8.6 ± 1.8	11.4 ± 2.3	t = -6.77	<0.001*
Retraction rate-incisors (mm/month)	0.82 ± 0.14	0.58 ± 0.11	t = 9.53	<0.001*
Overall space closure rate (mm/month)	0.77 ± 0.13	0.54 ± 0.11	t = 9.55	<0.001*
Leveling and alignment duration (months)	6.2 ± 1.4	6.5 ± 1.60	t = -0.99	0.302

Table 4 presents the extraction space closure efficiency and residual extraction space. The initial extraction space available at the start of treatment was comparable between the groups (p = 0.217). At the end of treatment, the en masse retraction group exhibited a significantly lower residual extraction space (0.42 ± 0.38 mm) than the two-step retraction group (0.61 ± 0.44 mm) (p = 0.018). Extraction space closure efficiency was significantly greater in the en masse retraction group (96.90 ± 2.80%) than in the two-step retraction group (95.40 ± 3.20%) (p = 0.011). In contrast, a significantly higher proportion of extraction space was utilized for incisor retraction in the two-step retraction group (79.30 ± 4.90%) than in the en masse retraction group (74.80 ± 5.60%) (p < 0.001). Anchorage loss accounted for a greater proportion of space consumption in the en masse retraction group (25.20 ± 5.60%) than in the two-step retraction group (20.70 ± 4.90%) (p < 0.001).

**Table 4:** Comparison of extraction space closure efficiency and residual extraction space between the study groups. Data presented as mean ± standard deviation (SD) or percentage (%), independent samples t-test used for intergroup comparisons, *p < 0.05 considered statistically significant, SD: standard deviation.

Parameter	En masse retraction (mean ± SD)	Two-step retraction (mean ± SD)	Test value	p-value
Extraction space available at start (mm)	13.60 ± 0.90	13.40 ± 0.80	t = 1.17	0.217
Residual extraction space at treatment completion (mm)	0.42 ± 0.38	0.61 ± 0.44	t = -2.31	0.018*
Extraction space closure efficiency (%)	96.90 ± 2.80	95.40 ± 3.20	t = 2.49	0.011*
Space used for incisor retraction (%)	74.80 ± 5.60	79.30 ± 4.90	t = -4.27	<0.001*
Space consumed by anchorage loss (%)	25.20 ± 5.60	20.70 ± 4.90	t = 4.27	<0.001*

Table 5 shows the dimensional changes in the transverse arch after treatment. The baseline intercanine, intermolar, and interpremolar widths were comparable between the groups (p > 0.05). The en masse retraction group showed a significant reduction in intercanine width (−0.58 ± 0.42 mm), whereas the two-step retraction group exhibited a slight increase (0.14 ± 0.38 mm) (p < 0.001). Similar findings were observed for intermolar width changes (−0.69 ± 0.54 mm vs. 0.08 ± 0.46 mm; p < 0.001) and interpremolar width changes (−0.44 ± 0.36 mm vs. 0.22 ± 0.31 mm; p < 0.001), indicating greater transverse arch constriction in the en masse retraction group.

**Table 5:** Comparison of transverse arch dimensional changes between the study groups. Data presented as mean ± standard deviation (SD), independent samples t-test used for intergroup comparisons, negative values indicate constriction and positive values indicate expansion, *p < 0.05 considered statistically significant, SD: standard deviation.

Parameter	En masse retraction (mean ± SD)	Two-step retraction (mean ± SD)	Test value	p-value
Intercanine width-pretreatment (mm)	33.8 ± 2.1	33.5 ± 2.3	t = 0.68	0.486
Intercanine width-post-treatment (mm)	33.2 ± 2.0	33.6 ± 2.1	t = -0.97	0.325
Change in intercanine width (mm)	-0.58 ± 0.42	0.14 ± 0.38	t = -8.98	<0.001*
Intermolar width-pretreatment (mm)	47.2 ± 2.8	47.0 ± 2.6	t = 0.37	0.701
Intermolar width-post-treatment (mm)	46.5 ± 2.7	47.1 ± 2.5	t = -1.15	0.208
Change in intermolar width (mm)	-0.69 ± 0.54	0.08 ± 0.46	t = -7.67	<0.001*
Interpremolar width-pretreatment (mm)	38.4 ± 2.3	38.2 ± 2.4	t = 0.42	0.644
Change in interpremolar width (mm)	-0.44 ± 0.36	0.22 ± 0.31	t = -9.823	<0.001*

Table 6 presents the intra-examiner reliability assessments. Excellent reliability was observed for all the evaluated variables, with ICC values ranging from 0.968 to 0.991. The highest reliability was observed for the space closure duration (ICC = 0.991), followed by the extraction space closure efficiency (ICC = 0.985) and maxillary incisor retraction measurements (ICC = 0.982). All ICC values were statistically significant (p < 0.001), confirming excellent measurement reproducibility and minimal intra-examiner errors throughout the study.

**Table 6:** Intra-examiner reliability assessment using the intraclass correlation coefficient. Data presented as intraclass correlation coefficient (ICC) with 95% confidence interval, ICC calculated using a two-way mixed-effects model with absolute agreement, ICC > 0.90 indicates excellent reliability, p < 0.05 considered statistically significant. ICC: intraclass correlation coefficient, CI: confidence interval.

Parameter	ICC	95% Confidence Interval	p-value
Incisor retraction (mm)	0.982	0.971-0.989	<0.001
Anchorage loss-first molar (mm)	0.976	0.962-0.985	<0.001
Anchorage loss-second premolar (mm)	0.974	0.959-0.983	<0.001
Space closure duration (months)	0.991	0.986-0.994	<0.001
Retraction rate-incisors (mm/month)	0.979	0.967-0.987	<0.001
Extraction space closure efficiency (%)	0.985	0.976-0.991	<0.001
Residual extraction space (mm)	0.972	0.956-0.982	<0.001
Change in intercanine width (mm)	0.969	0.951-0.980	<0.001
Change in intermolar width (mm)	0.971	0.954-0.982	<0.001
Change in interpremolar width (mm)	0.968	0.950-0.980	<0.001

## Discussion

The present retrospective comparative study evaluated the clinical outcomes of en masse retraction and two-step retraction mechanics in patients with Class I bimaxillary protrusion treated with first premolar extraction. The findings demonstrated that both treatment modalities were effective in achieving anterior tooth retraction and extraction-space closure. However, significant differences were observed in anchorage preservation, treatment efficiency, extraction space utilization, and transverse arch dimension changes.

The baseline demographic and pretreatment characteristics were comparable between the two groups, with no statistically significant differences in age, sex distribution, skeletal pattern, overjet, overbite, or treatment duration. This comparability strengthens the internal validity of the study and suggests that the observed differences in treatment outcomes were primarily attributable to the retraction mechanics employed rather than the baseline disparities between the groups.

With respect to anterior tooth movement, both treatment approaches achieved comparable amounts of maxillary incisor retraction. Although the en masse retraction group demonstrated slightly greater mean incisor retraction, the differences were not statistically significant. These findings are consistent with those reported by Felemban et al. [8], who observed that both retraction strategies could produce similar amounts of anterior tooth movement when adequate extraction space was available. The comparable retraction outcomes suggest that both techniques can effectively address dentoalveolar protrusion and improve facial esthetics in patients with bimaxillary protrusion.

A major finding of the present study was the significantly greater anchorage loss observed in the en masse retraction group. Mesial movement of both the first molars and second premolars was substantially higher than that in the two-step retraction group. This finding may be explained by the biomechanical characteristics of en masse retraction, in which larger forces are applied simultaneously to retract the entire anterior segment of the dental arch. These forces increase the load on the posterior anchorage units and consequently promote greater mesial movement of the posterior teeth [10]. Similar findings were reported by Felemban et al. [8] and Vishva et al. [11], who demonstrated increased anchorage demands during en masse retraction compared with sequential canine and incisor retraction. The significantly higher retraction-to-anchorage loss ratio observed in the two-step retraction group further supports the superior anchorage efficiency of this approach.

The findings of the present study differ from those of Rizk et al. [3], who reported reduced anchorage loss and shorter treatment duration with en masse retraction. However, most studies included in their systematic review utilized mini-implants or skeletal anchorage, which substantially enhanced anchorage control. In contrast, the present study evaluated conventional retraction mechanics without skeletal anchorage, which may explain the greater anchorage loss observed in the en masse retraction group.

Despite the increased anchorage loss, the en masse retraction group demonstrated significantly faster treatment mechanics. The space closure duration was approximately three months shorter than that observed in the two-step retraction group, and the rates of incisor retraction and overall space closure were significantly greater. These findings are in agreement with previous investigations by Upadhyay et al. [12], Felemban et al. [8], Rizk et al. [3], and Al-Sibaie and Hajeer [13], who reported that simultaneous retraction of the anterior segment reduces the number of treatment stages and enhances overall treatment efficiency. The elimination of a separate canine retraction phase likely contributes to the shorter treatment duration associated with en masse mechanics.

The present study also demonstrated superior extraction space closure efficiency in the en masse retraction group. The residual extraction space at the end of treatment was significantly lower, and the overall efficiency of space closure was significantly greater. These findings suggest that continuous space closure mechanics may facilitate more complete utilization of extraction spaces [14]. However, a greater proportion of extraction space was consumed by anchorage loss in the en masse group, whereas a significantly larger proportion of space was used for anterior tooth movement in the two-step retraction group. This observation highlights the clinical trade-off between treatment efficiency and the preservation of anchorage. While en masse retraction offers faster space closure, part of the extraction space may be lost because of posterior tooth mesialization.

The findings of the present study are also not in agreement with those of Basha et al. [15], who reported that mini-implant-supported en masse retraction provided absolute anchorage and significantly reduced anchorage loss compared with conventional molar anchorage. This discrepancy may be attributed to the use of skeletal anchorage in their study, which effectively eliminated posterior tooth movement during retraction. In contrast, the present study evaluated conventional retraction mechanics without mini-implants, making some degree of anchorage loss inevitable, particularly during en masse retraction.

Another noteworthy finding was the greater transverse arch constriction observed in the en masse retraction group. Significant reductions in intercanine, intermolar, and interpremolar widths were observed, whereas slight expansion or maintenance of transverse dimensions was noted in the two-step retraction group. These findings may be attributed to the inward component of the retraction forces generated during simultaneous anterior segment movement, resulting in narrowing of the dental arch. Similar transverse dimensional reductions during extraction treatment have been reported by Herzog et al. [16] and Elias et al. [17]. A recent evidence-based review by Arsic et al. [18] highlighted that premolar extraction treatment tends to reduce arch width compared with non-extraction therapy, although the available evidence is inconsistent. Excessive arch constriction may influence smile esthetics, buccal corridor appearance, and long-term post-treatment stability, emphasizing the importance of monitoring transverse dimensions during space closure.

From a clinical perspective, the results suggest that en masse retraction may be preferred when treatment efficiency and shorter space closure duration are the primary objectives. Conversely, two-step retraction may be advantageous in patients requiring maximum anchorage preservation, particularly when skeletal anchorage devices are not used. Therefore, the choice of retraction strategy should be individualized according to the patient's anchorage requirements, treatment objectives, and biomechanical considerations.

The present study had certain limitations. Its retrospective design inherently limits the control over treatment variables and may introduce selection bias. Variations in operator technique, bracket prescription, force application protocols, and patient compliance could not be completely standardized. The study was conducted at a single institution and included only patients with Class I bimaxillary protrusion, which may limit the generalizability of the findings to other malocclusion types and treatment protocols. Furthermore, the analysis was based on conventional plaster study models rather than three-dimensional digital models, which may have limited the precision of some measurements. Cephalometric variables related to incisor inclination, skeletal changes, and soft tissue profile alterations were not evaluated. In addition, advanced imaging modalities such as cone-beam computed tomography (CBCT) were not utilized; therefore, three-dimensional tooth movement, alveolar bone changes, and root resorption could not be evaluated. Long-term post-treatment stability, periodontal outcomes, and patient-reported outcomes were not investigated. Parameters such as pain perception, treatment-related discomfort, oral function, quality of life, and patient satisfaction may provide valuable insights into the overall patient experience associated with different retraction mechanics and should be considered in future studies. The changes were evaluated only in the maxillary arch. Future prospective randomized controlled trials incorporating skeletal anchorage systems, digital model analysis, cephalometric and CBCT assessments, root resorption evaluation, comprehensive patient-reported outcome measures, and long-term follow-up are recommended to provide a more comprehensive comparison of these retraction modalities.

## Conclusions

Both en masse and two-step retraction mechanics were effective in achieving maxillary incisor retraction and extraction space closure in patients with Class I bimaxillary protrusion. En masse retraction demonstrated significantly faster space closure, higher retraction rates, and greater extraction space closure efficiency; however, it was associated with increased anchorage loss and greater transverse arch constriction. In contrast, two-step retraction provided superior anchorage preservation and more favorable transverse dimensional stability. Therefore, the selection of retraction mechanics should be based on the individual treatment objectives, anchorage requirements, and biomechanical considerations.
